# Cell protective effects of vitamin C against oxidative stress induced by ciprofloxacin on spermatogenesis: involvement of cellular apoptosis

**DOI:** 10.3389/fcell.2025.1489959

**Published:** 2025-03-20

**Authors:** Nihal A. Ibrahim, Manal A. Buabeid, Kadreya E. Elmorshedy, El-Shaimaa A. Arafa

**Affiliations:** ^1^ Department of Clinical Sciences, College of Pharmacy and Health Sciences, Ajman University, Ajman, United Arab Emirates; ^2^ Center of Medical and Bio-allied Health Sciences Research, Ajman University, Ajman, United Arab Emirates; ^3^ Fatima College of Health Sciences, Department of Pharmacy, Abu Dhabi, United Arab Emirates; ^4^ College of Medicine, Anatomy Department, King Khaled university, Abha, Saudi Arabia; ^5^ College of Medicine, Anatomy Department, Tanta University, Tanta, Egypt

**Keywords:** spermatogenesis, ciprofloxacin, apoptosis, vitamin C, oxidative stress

## Abstract

**Introduction:**

Ciprofloxacin (CPFX), a second-generation fluoroquinolone, is widely used as an anti-infective agent for genitourinary tract infections due to its broad-spectrum efficacy against gram-positive and gram-negative organisms. Although CPFX is considered safe at therapeutic doses, recent evidence suggests its potential biological toxicity, particularly affecting testicular histology and function. This study aimed to investigate the effects of CPFX on testicular structure and function and to evaluate the protective role of vitamin C.

**Methods:**

Forty adult male albino rats were divided into four groups: control, CPFX-treated, vitamin C-treated, and CPFX combined with vitamin C-treated. After 60 days of treatment, blood samples were collected for hormonal assays, while testicular and epididymal tissues were analyzed using light and electron microscopy. Oxidative stress markers, including malondialdehyde (MDA), glutathione (GSH), and catalase (CAT) enzyme activity, were assessed. Statistical analyses were conducted using SPSS software.

**Results:**

Confocal microscopy of the CPFX-treated group revealed significant reductions in germ cell populations within seminiferous tubules, accompanied by severe apoptosis and degenerative epithelial changes. Morphometric analysis confirmed a decrease in tubular diameter and epithelial height, degeneration of spermatogenic cells, and detachment of apoptotic cells from the basement membrane. CPFX treatment significantly reduced testosterone levels and induced variable changes in gonadotropin hormones (LH and FSH). Co-administration of vitamin C with CPFX restored normal testicular morphology, preserving seminiferous tubule integrity and maintaining spermatogenic cell populations and spermatozoa within the lumen.

**Discussion and Conclusion:**

Vitamin C supplementation effectively mitigated CPFX-induced oxidative stress by significantly reducing MDA levels and enhancing antioxidant defenses, including increased GSH content and CAT enzyme activity. These findings highlight the therapeutic potential of vitamin C in reversing CPFX-induced testicular toxicity by alleviating oxidative stress and restoring testicular function.

## 1 Introduction

Ciprofloxacin (CPFX), a synthetic fluoroquinolone antibiotic, is effective against multiple microbial infections and is approved in most countries. CPFX is a fluoroquinolone antibiotic that acts on the bacterial DNA gyrase enzyme and has been approved for widespread use in most countries worldwide due to its ability to successfully eliminate a broad spectrum of microbial pathogens, especially Gram-negative organisms. Nevertheless, the preceding meta-analysis studies have reported a recent decline in semen quality, which testifies to environmental factors, including chemical exposure and medication use (Sharma et al., 2017; Kheirandish et al., 2020). Although CPFX has been proven to be a highly effective antibacterial agent against various bacterial strains, it has been proved that short-term exposure can also have detrimental effects on the male reproductive system and, therefore on male fertility, which raises concerns about CPFX toxicity (Abd-Allah et al., 2000; Demir et al., 2007). Potential side effects include gastrointestinal disturbances, which include nausea vomiting, diarrhea, dizziness, and, sometimes, convulsions—common to all quinolones. In particular, CPFX has been related to several additional side-effects on the male genital system. Research studies have indicated that quinolones have the potential to interfere with normal human DNA and stimulate oxidative DNA damage (Mandell and Tillotson, 2002; Rusu et al., 2023).

Spermatogenesis is the process of sperm development and maturation in mammals, which is composed of two main phases: (i) spermatogenesis, during which spermatogonia undergo successive mitotic divisions to form spermatocytes, which then divide meiotically to form round spermatids, and (ii) spermiogenesis, during which round spermatids undergo morphological changes, thereby maturing into elongated spermatids and into spermatozoa that are adapted for fertilization. Leydig and Sertoli cells are ultimately necessary for spermatogenesis and cellular metabolism, thus playing a major role in intratesticular endocrine activity. The worst situation occurs when CPFX affects testicular tissue stem cells and their physiological connection with the germinal epithelium (GE). Alterations in cytoplasmic biochemistry within the GE and inflammation impacting spermatogenesis and spermiogenesis processes remain poorly understood (Zobeiri et al., 2013; Adamczewska et al., 2022).

The apoptotic effects of CPFX on sperm cells and testicular tissue are linked to the mitochondrial pathway. CPFX induces apoptosis and inhibits cell growth in certain eukaryotic cells, with these effects showing a dose-dependent relationship. Additionally, CPFX significantly increases serum acid phosphatase activity. These findings, along with histopathological changes, shed light on the potential mechanisms underlying testicular dysfunction caused by CPFX (Khaki, 2015).

The complex process of spermatogenesis depends on growth factors and hormones that function through endocrine and paracrine pathways. The main somatic cells found in the seminiferous tubules are called Sertoli cells (SCs), which are the primary regulators of spermatogenesis. As each SC supports a specific number of germ cells, the final number of SCs determines the sperm production capacity. Similarly, sex hormones can control SC growth and are important regulators of spermatogenesis (Shah et al., 2021).

Administration of CPFX decreases testicular weight depending on the dosages and the time of exposure to ciprofloxacin. Furthermore, CPFX reduces the sperm count and the mobility of the motile sperm in male guinea pigs, depending on the given dose and the duration of the experiment; the effects of CPFX on the shape and size of sperm are also dose-dependent. Notably, CPFX produces a significant dose- and time-dependent decrease in testosterone (Elias and Nelson, 2012).

Prolonged use of CPFX has the effect of changing some of the membrane features of sperms, enabling reduced movement, especially their fast-progressive motion. Moreover, leukocytosis and/or lymphocytopenia have been detected, along with focal necrotic degeneration, inflammation, and variable degrees of decreased or damaged germ cells in the testes in patients with COVID-19, implying that they might develop orchitis (Bian and COVID-19 Pathology Team, 2020; Yang et al., 2020).

Vitamin C or ascorbic acid is a water-soluble antioxidant that protects the body from major hazards caused by molecule chain reactions. Of the non-enzymic antioxidant types present in the body, this is a key compound essential for the reversal of oxidative breakdown and is relatively cheap and readily available (Naidu, 2003; Magdy et al., 2016).

Increased release of reactive oxygen species (ROS) is a regular occurrence because they are the byproducts of cellular metabolism, but the sustained abundance of ROS can lead to an increased level of oxidative stress. This results in the generation of hydroxyl free radicals and other toxic agents that bring about lipid peroxidation, protein, and DNA modifications, leading to cell death (Yin et al., 2022). Free radicals, also known as ROS, are continuously generated within the human body and can cause oxidative stress when the body’s defenses against free radicals are overpowered.

Literature has indicated that when vitamins C and E are administered independently as antioxidants, they provide some protection to the liver, kidneys, and testes against drug-induced toxicity. Unfortunately, they do not provide total protection from such stresses, in particular from liver tissue damage. According to the findings of recent animal experimental studies, vitamin C increases the activity of antioxidant enzymes and significantly lowers testicular antioxidant state and malondialdehyde (MDA), a marker of oxidative stress, when compared to the test group. Supplementation of vitamin C as an antioxidant in a dose-dependent manner may improve sperm quality in men. Due to its relatively low cost in comparison to other antioxidants and its low toxicity, oral administration of this compound reduces the negative impacts triggered by several xenobiotics, according to Karmmon et al. (2011), Uchendu et al. (2012), and Chambial et al. (2013). Vitamin C also plays a role in protecting organisms from the toxic effects of xenobiotics and offers some protection against heat shock injuries. By preserving tissue architecture, delaying protein oxidation, and increasing tissue stability, supplemental ascorbic acids aid in the mitigation of the detrimental impacts of heat stress on various tissues (Zhao et al., 2022).

**DIAGRAM 1:**
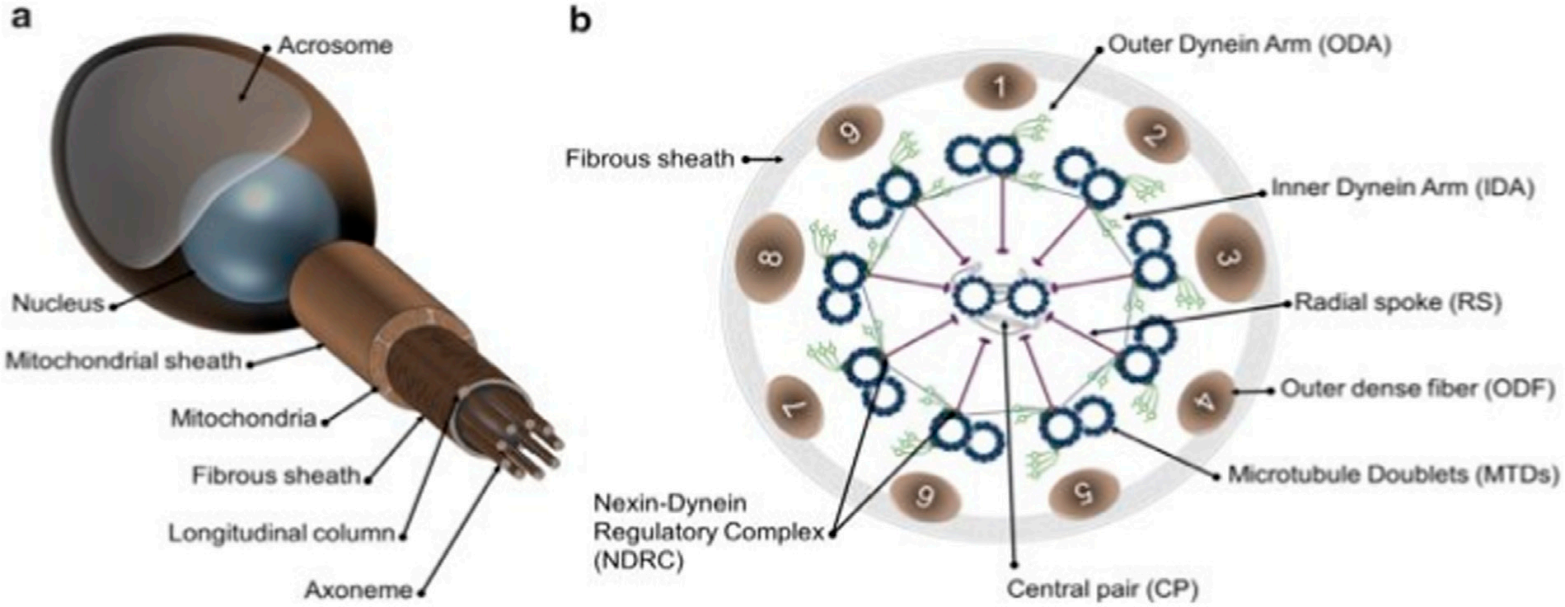
Schematic representation of mammalian spermatozoa and flagellum structure. **(A)**: overall view of the spermatozoa showing the main head and flagellum structures and compartments. **(B)**: cross section from the principal piece of the flagellum showing the organization of the axoneme. Adapted from Toure et al. (2021).

The main features of the flagellum and spermatozoa in mammals are shown in Diagram 1A, B As depicted on the left side, a non-structural feature of a human constitutional sperm includes compartments and components of the sperm’s flagella. The main parts are the head, axonem, inner and outer dynein arms (IDA and ODA), central pair (CP), radial spokes (RS), microtubule doublets (MTD), nexin–dynein regulatory complex (N-DRC), fibrous sheath (FS), and outer dense fiber. The diagram on the far right depicts a cross-sectional view of the axoneme and reveals details of its internal structure. According to Toure et al. (2021), the flagellum is vital to the mobility of spermatozoa, enabling sperm cells to move in the female reproductive system to reach the egg for fertilization. Thus, certain structures are highly complex and tightly regulated to perform their functions well.

## 2 Material and methods

### 2.1 Animals and study design

Forty male Dawley rats were selected and divided into four groups.

#### 2.1.1 Group I (control sham group)

This group consisted of 10 rats that received 0.5% carboxymethyl cellulose (CMC) orally for 60 successive days (Reagan-Shaw et al., 2007).

#### 2.1.2 Group II (CPFX-treated group)

This group consisted of 10 rats that received CPFX at a dose of 12.5 mg/kg, liquefied in 0.5% CMC, orally via a gastric tube once every day for 60 successive days. Doses of CPFX were equivalent to human therapeutic doses (Hou et al., 2022).

#### 2.1.3 Group III (vitamin C-treated group)

This group consisted of 10 rats that received vitamin C (100 mg/kg) liquefied in 0.5 mL of purified water via an esophageal tube once every day for 60 days (El Kotb et al., 2020; Baloglu et al., 2023; Aruwa et al., 2024).

#### 2.1.4 Group IV (CPFX and vitamin C-treated group)

This group consisted of 10 rats that received CPFX and vitamin C (100 mg/kg) liquefied in 0.5 mL of purified water via an esophageal tube once every day for 60 days.

On the 60th day, the testicular and epididymal tissues of all groups were collected and prepared for light and electron microscopic examinations (Mojoyinola et al., 2023).

### 2.2 Tissue processing for light microscopy

Following 4 h of fixation in 10% formaldehyde, tissue specimens were dehydrated in increasing concentrations of alcohol, followed by paraffin-embedding. The tissues were then cut into 5-*µ*m-thick slices. Hematoxylin and eosin and Mason’s trichrome staining were performed to examine the pathological alterations in the rat testes using a light microscope (Ibrahim et al., 2022; Da Silva et al., 2023).

### 2.3 Morphometric study

Seminiferous tubules’ diameter and epithelial height were measured using the following tools and methods:

1. Images of the seminiferous tubules were captured using an Olympus digital camera at a constant magnification. The ImageJ tool was then utilized to measure the distance between specific points within the captured images, ensuring accuracy.
2. Each rat was evaluated using five slides, with each slide containing five random fields for analysis. This allowed for the comprehensive examination of the specimens.
3. In the five slides, at least five measurements (in micrometers, µm) for each field were done manually using ImageJ software (version:1.50i) (Abarikwu et al., 2020).

### 2.4 Electron microscopic studies

Freshly cut, small (1 mm) portions of treatment and control tissues were fixed in 3% glutaraldehyde (pH7.4) in phosphate buffer and then post-fixed in 2% osmium tetroxide (OsO4) in phosphate buffer. The tissues were dehydrated using escalating ethanol concentrations after fixation. After that, Araldite resin was used to embed them. Using an ultramicrotome, ultrathin sections were cut, and uranyl acetate soaked in 70% ethanol and lead citrate was used to stain them. Using a JEOL transmission electron microscope, ultrathin sections of rat testis were examined at Cairo University’s Faculty of Agriculture’s Central Research Park (Tousson et al., 2011; Ibrahim et al., 2023).

### 2.5 Hormonal assay

Blood samples from all animals in the four groups were collected after scarification from the inferior vena cava. Following centrifugation, samples were assessed for serum levels of LH, FSH, and testosterone by ELISA technique using commercial ELISA kits, according to the manufacturer’s instructions, at AlSafwa laboratory, Egypt (Altwaijry et al., 2020; Kheirandish et al., 2020).

### 2.6 Assessment of oxidative stress in testicular tissue

Testicular tissue was homogenized in cold phosphate-buffered saline (20% w/v) and centrifuged for 15 min at 2000 g. The supernatant was used for determining various biochemical parameters, such as MDA, glutathione (GSH), and catalase (CAT) activity. Oxidative stress in the testicular tissue was assessed using established methods. The levels of MDA and GSH were determined using Ellman’s method (Ellman, 1959) and Uchiyama and Mihara’s method (Mihara &Uchiyama, 1978), respectively. The activity of CAT was measured following Aebi’s protocol (Aebi, 1974).

### 2.7 Statistical analysis

Data were analyzed using the Statistical Program for Social Science (SPSS) version 24.0. Quantitative data were expressed as the mean ± standard deviation (SD). A one-way analysis of variance (ANOVA) was used to compare more than two means. A *p-*value of less than 0.05 was considered statistically significant (Alfadda et., 2022; Zhang et al., 2024).

#### 2.7.1 Ethics statement

Research was approved by the Ethical Committee of Ajman University under ethical clearance number: P-F-A-11-Oct. The study was conducted in compliance with the university’s ethical guidelines and international standards.

## 3 Results

### 3.1 Light microscopic studies

**FIGURE 1:**
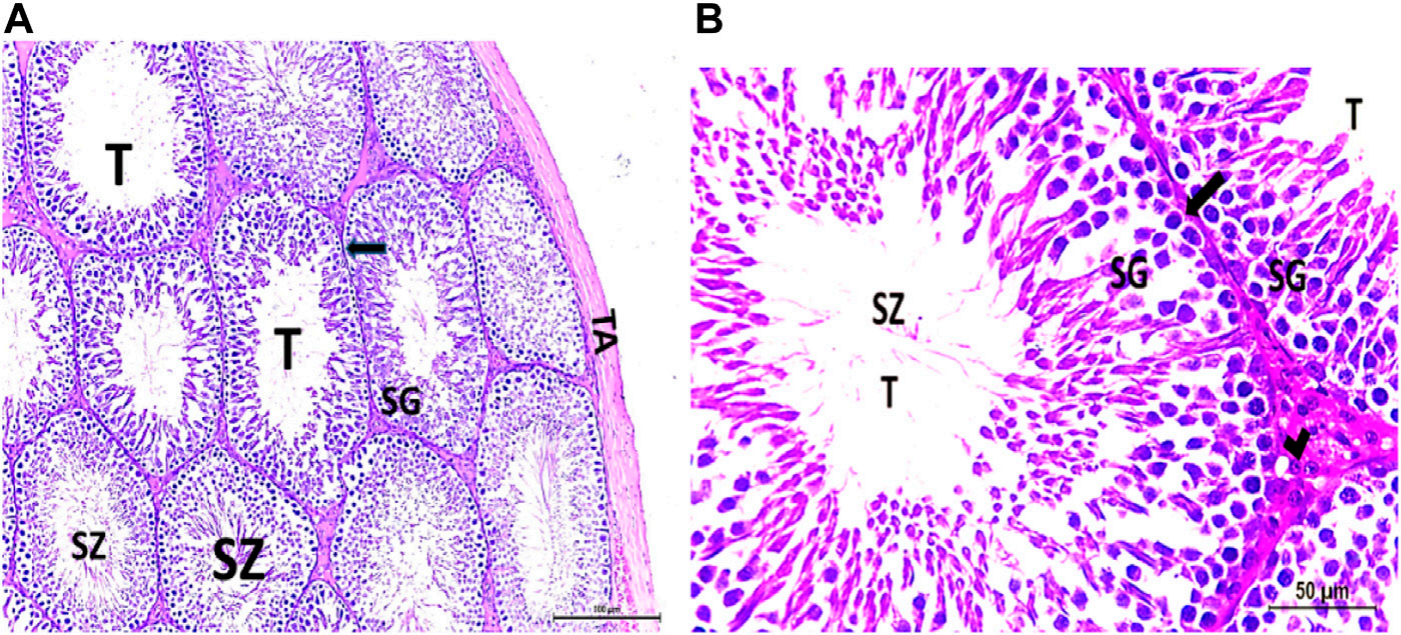
**(A, B)** Photomicrograph of a control rat testis covered by the tunica albuginea (TA). Apparently normal, oval to rounded seminiferous tubules (T) with a regular contour are observed. Spermatozoa (SZ) are filling the lumina of the tubules. Spermatogenic (SG) cells resting on the intact basement membrane (arrows) and interstitial Leydig cells (arrow head) are clearly observed (Hx and E × 200 and 400).

**FIGURE 2:**
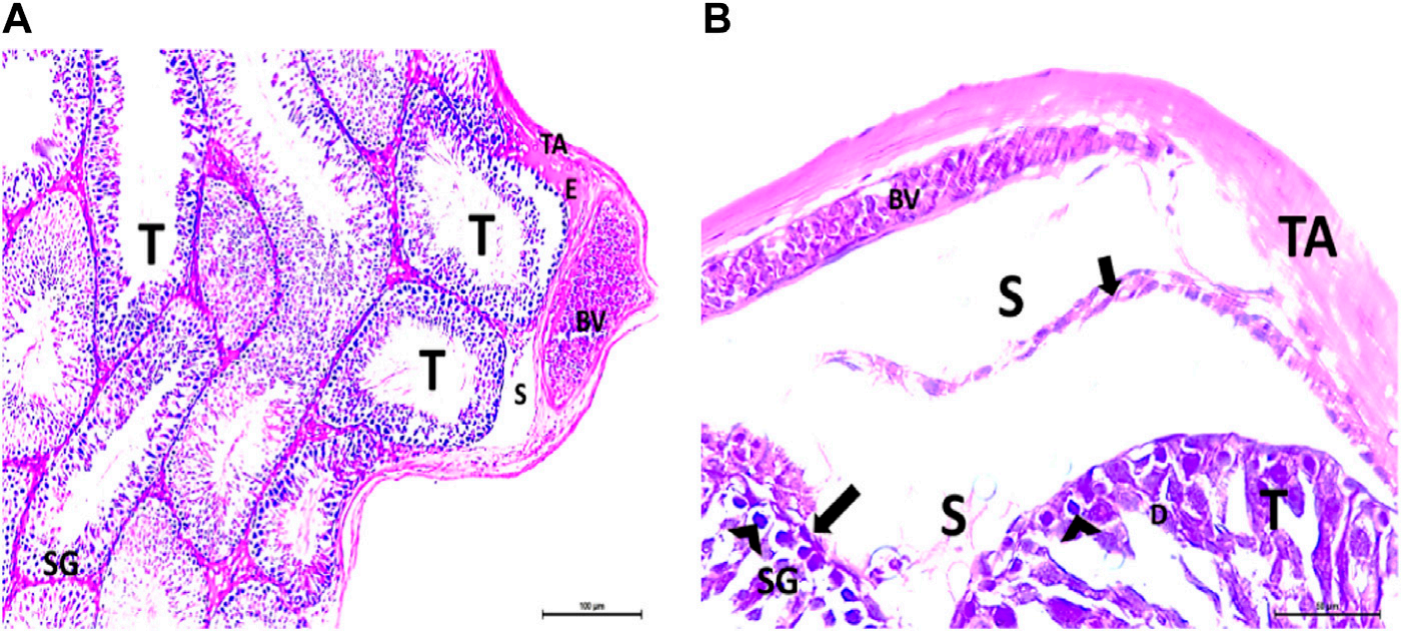
**(A, B)** Photomicrographs of the rat testis of the CPFX-treated group showing an irregular contour of seminiferous tubules (T) with a zone of separation (S) between them and TA. Congested and dilated blood vessels (BVs) beneath the TA are noticed. Degeneration (D) of spermatogenic epithelial series (SG) with apoptotic cells (arrow heads) resting on a detached basement membrane (arrow) (HX and E × 200 and400).

The testis of control and vitamin C-treated rats is covered by the tunica albuginea. Apparently normal, oval to rounded seminiferous tubules with a regular contour are observed, with spermatozoa filling the lumina of the tubules. Spermatogenic cells resting on an intact basement membrane (BM) and interstitial Leydig cells are clearly visible (Figures 1A, B). In the CPFX-treated group, seminiferous tubules display irregular contours with a zone of separation between them and the tunica albuginea. Congested and dilated blood vessels beneath the tunica albuginea are noticeable. Degeneration of the spermatogenic epithelium, with apoptotic cells resting on a detached basement membrane, is evident. Testicular slices also display seminiferous tubules lined with chains of spermatogenic cells, with few fragmented sperms appearing in the dilated lumens. Tubules are surrounded by a thickened basement membrane with interrupted zones. The interstitial spaces between the tubules show edema and cellular infiltrates around blood vessels (Figures 2A, B). The CPFX- and vitamin C-treated rat testis shows normal, oval to rounded seminiferous tubules with a regular contour. Spermatozoa fill the lumina of the tubules. Spermatogenic cells rest on an intact basement membrane, although some appear slightly detached from it. Interstitial exudate is also visible (Figures 5A, B). Photomicrographs of control and vitamin C-treated epididymis show normal spermatozoa inside a ciliated epididymal epithelium, with a stromal blood vessel present (Figures 6A, 7A). The CPFX-treated epididymis shows degenerated spermatozoa inside a vacuolated epididymal epithelium with cilia loss (Figure 6B). Meanwhile, CPFX- and vitamin C-treated epididymis shows partial recovery, with spermatozoa observed inside a ciliated epididymal epithelium (Figure 7B).

#### 3.1.1 Testis

##### 3.1.1.1 Control group

Figures 1A, B

##### 3.1.1.2 CPFX-treated group

Figures 2A,B, 3A

**FIGURE 3:**
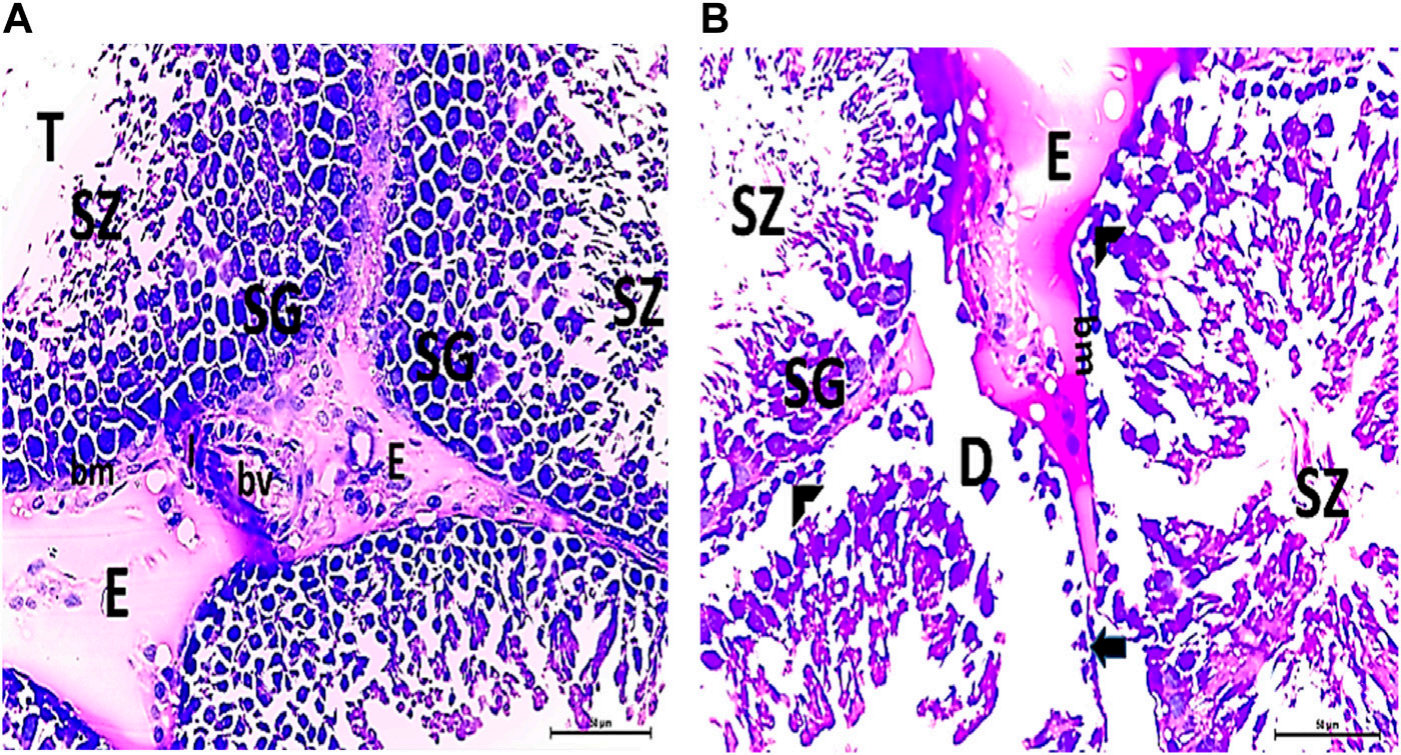
**(A, B)** Photomicrographs of CPFX-treated testicular slices displaying seminiferous tubules (T) lined with chains of spermatogenic cells (SG) with few fragmented irregular sperms appearing in the dilated lumens (SZ). Tubules (T) are surrounded by a thickened BM with interrupted zones (arrow). The interstitial spaces in-between the tubules show edema (E) with cellular infiltrate (I) around the blood vessel (HX and E × 400).

##### 3.1.1.3 Vitamin C-treated group

Figures 4A, B

**FIGURE 4:**
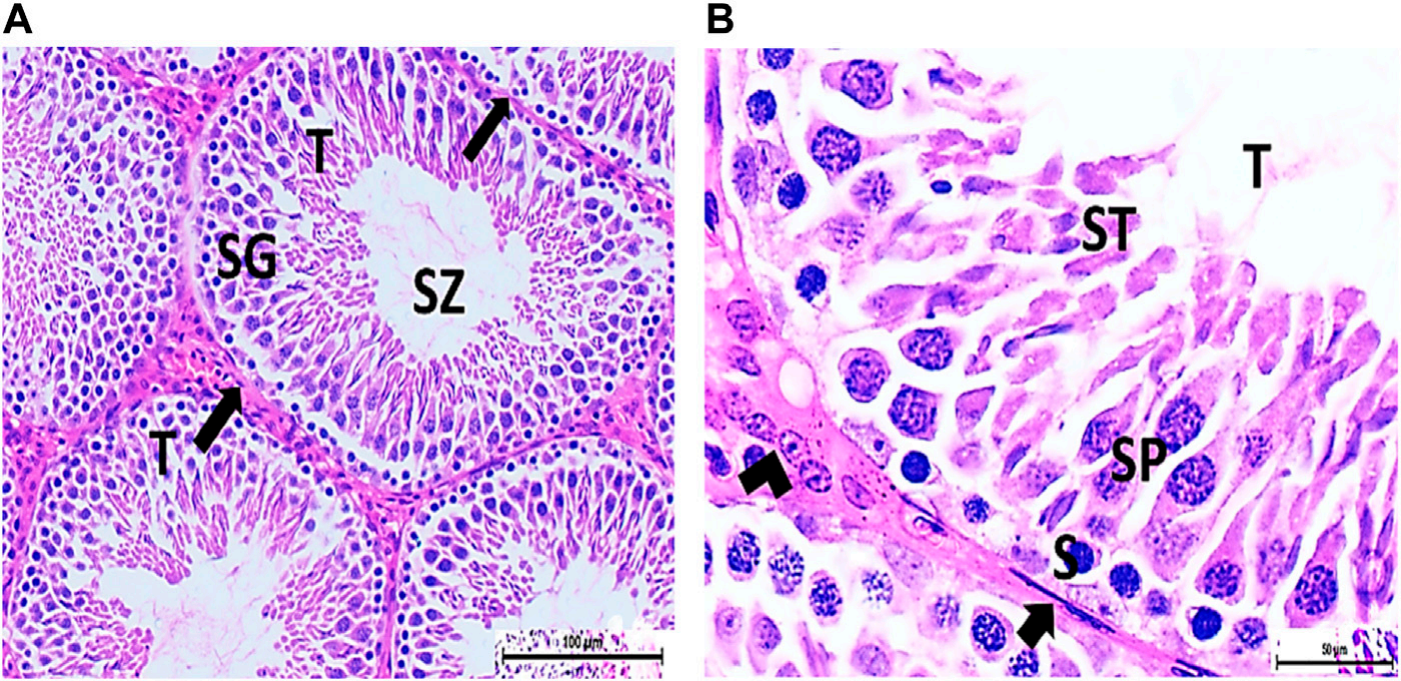
**(A)** Photomicrograph of vitamin C-treated rat testis showing normal, oval to rounded seminiferous tubules (T) with a regular contour. SZ are filling the lumina of the tubules. Spermatogenic cells resting on the intact basement membrane (arrows) are observed (HX and E × 200). **(B)** Magnified photomicrograph of the same group showing normal seminiferous tubules (T) with a regular contour. Spermatids (ST) are filling the lumina of the tubules. Spermatogonia (S) resting on the intact basement membrane (arrows) giving rise to spermatocytes (SP) are seen. Interstitial cells of Leydig with vesicular nuclei (arrow head) are visible (HX and E × 400).

##### 3.1.1.4 CPFX- and vitamin C-treated group

Figures 5A, B

**FIGURE 5:**
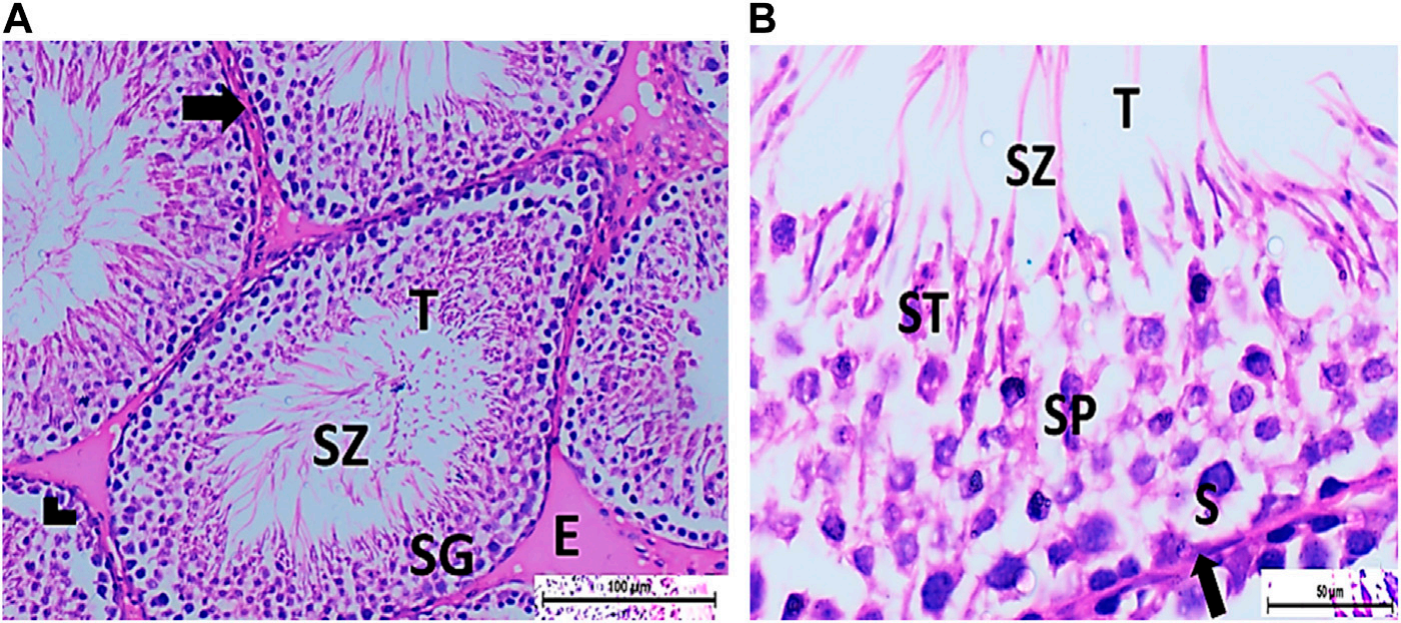
**(A)** Photomicrograph of CPFX- and vitamin C-treated rat testis showing normal, oval to rounded seminiferous tubules (T) with regular contour. Spermatozoa (SZ) are filling the lumina of the tubules. Spermatogenic cells resting on the intact basement membrane (arrows) are observed with some slight detachment (arrow head). Interstitial exudate (E) is also visible (HX and E × 200). **(B)** Photomicrograph of CPFX- and vitamin C-treated rat testis showing apparently normal seminiferous tubules (T) with a regular contour. Spermatids (ST) and SZ are filling their lumina. Spermatogonia (S) resting on intact basement membrane (arrows) giving rise to spermatocytes (SP) are seen. (HX and E × 400).

#### 3.1.2 Epididymis

Figures 6, 7

**FIGURE 6:**
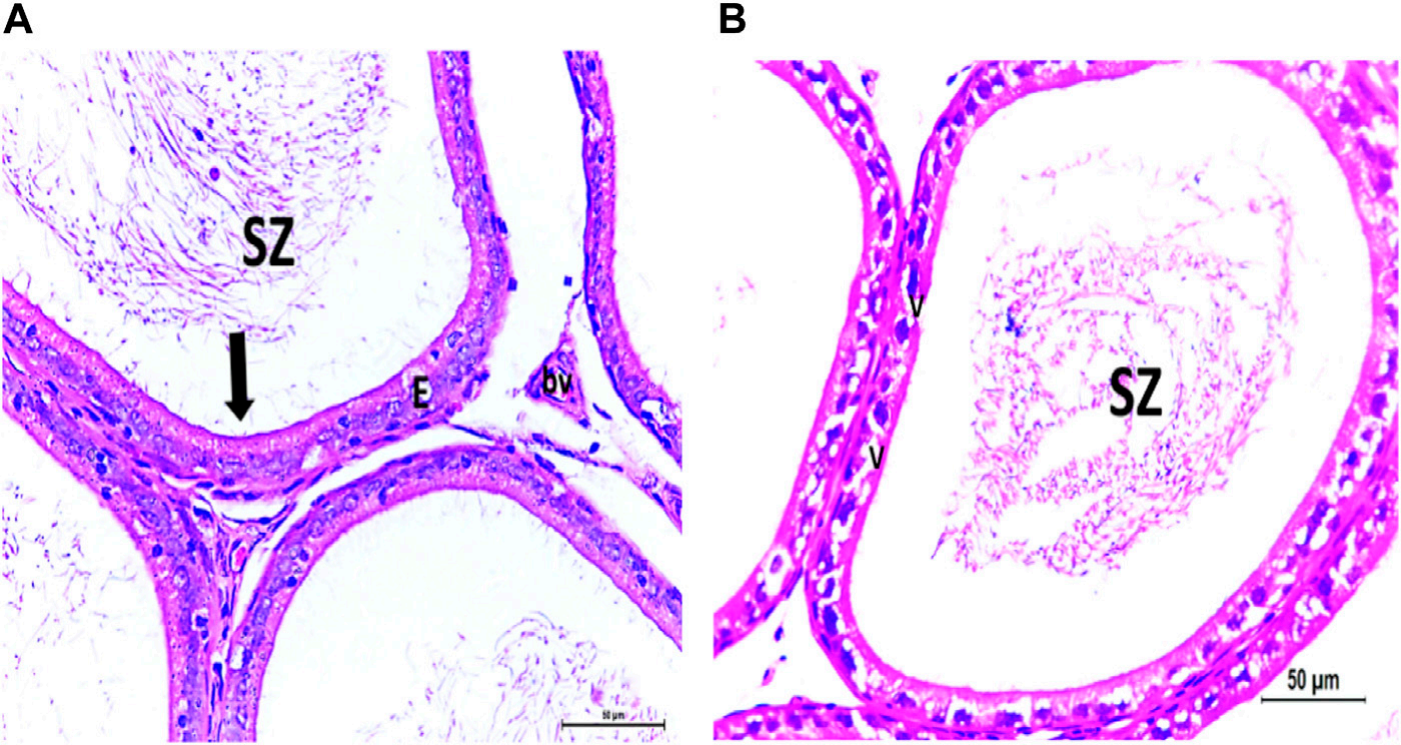
**(A)** Photomicrograph of control epididymis showing normal SZ inside the ciliated (arrow) epididymal epithelium (E). A stromal blood vessel (bv) is noticed. **(B)** Photomicrograph of CPFX epididymis showing degenerated SZ inside the vacuolated (V) epididymal epithelium with cilia loss.

**FIGURE 7:**
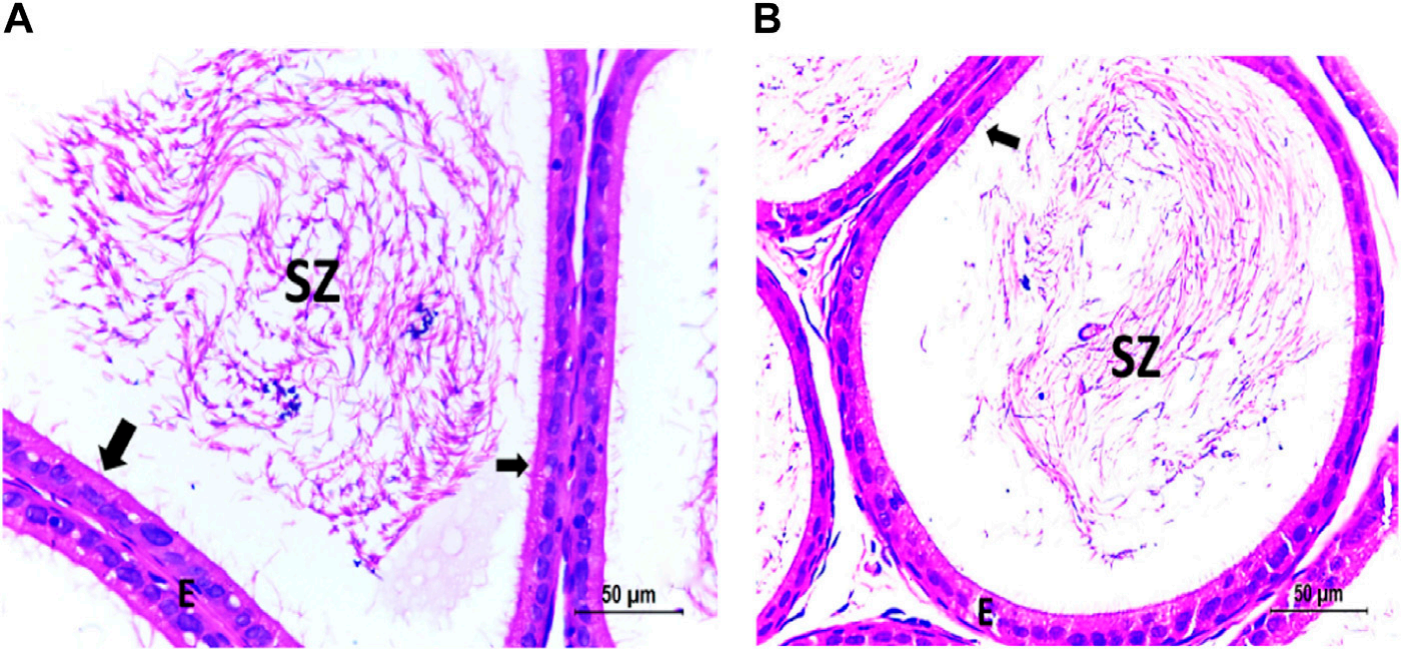
**(A)** Photomicrograph of vitamin C-treated epididymis showing normal SZ inside the ciliated (arrow) epididymal epithelium (E). **(B)** Photomicrograph of CPFX- and vitamin C treated epididymis showing partially recovered SZ inside the ciliated (arrow) epididymal epithelium (E).

### 3.2 Morphometric studies

#### 3.2.1 Semi-thin testicular sections

**FIGURE 8:**
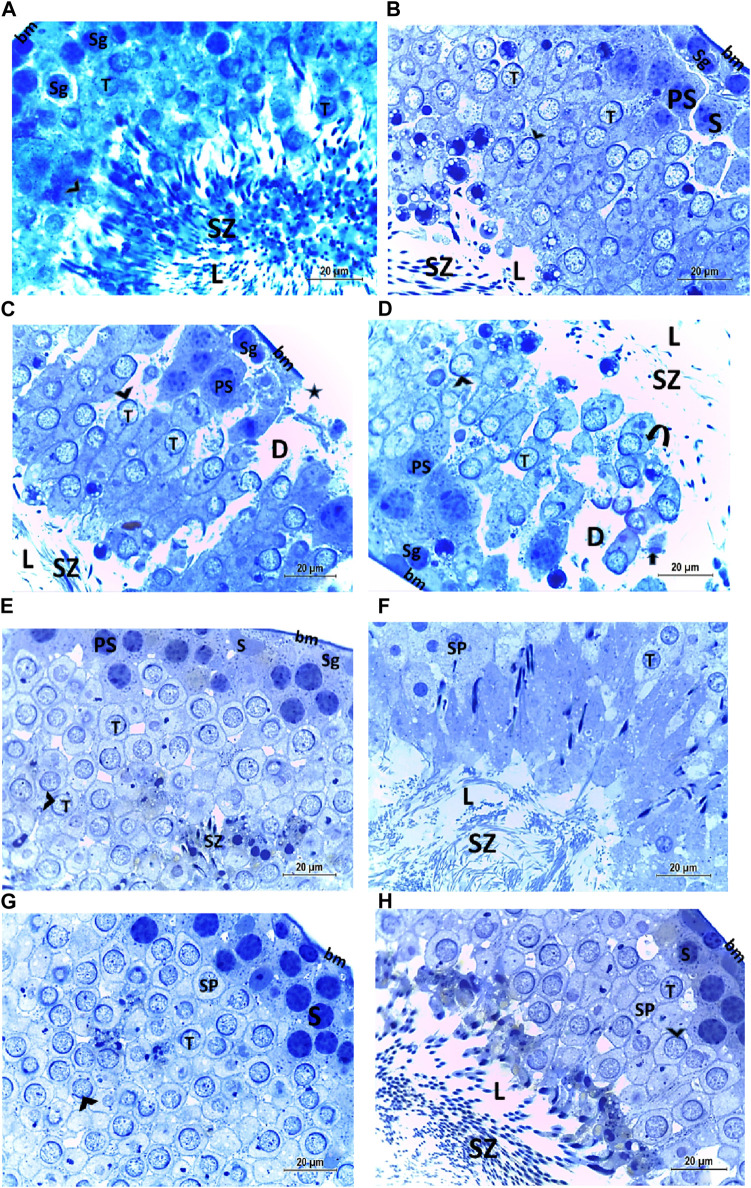
**(A, B)** Histological structure of control rat testis in toluidine blue-stained semi-thin sections showing seminiferous tubules with a normal shape and arrangement of spermatogenic cells (Sg) resting on the intact basement membrane (bm); large primary spermatocytes (PS) in different stages of meiotic division, round spermatids (T) with acrosomal caps (arrowhead), and numerous mature sperms (SZ) are observed inside the tubular lumen (L). **(C, D)** Histological structure of a testis from a rat treated with CPFX, stained with toluidine blue, and viewed using semi-thin sections reveals a reduction in the diameter of seminiferous tubules (D) and the presence of degenerated zones (arrows) among spermatogenic cells (SG) resting on disrupted (star) basement membranes. The tubular lumens (L) are filled with apoptotic SZ. Some early rounded spermatids (T) exhibit disrupted nuclear membranes (arrow heads), and multi-vesicular giant cells (curved arrows) with large-sized nuclei (arrowheads) are observed near the tubular lumens. **(E, F)** Histological structure of vitamin C-treated rat testis in toluidine blue-stained semi-thin sections showing seminiferous tubules with a normal shape and arrangement of Sertoli cells (S) with triangular shaped nuclei, spermatogenic cells (Sg) resting on intact basement membrane (bm), large primary spermatocytes (PS) in different stages of meiotic division, round spermatids (T) with acrosomal caps (arrowhead), and numerous mature SZ inside the testicular lumen (L). **(G, H)** Histological structure of CPFX- and vitamin C-treated rat testis in toluidine blue-stained semi-thin sections showing seminiferous tubules with a normal shape and arrangement of spermatogenic cells (Sg) resting on the intact basement membrane; large primary spermatocytes (PS) in different stages of meiotic division, numerous round spermatids (T) with acrosomal caps (arrowheads); and numerous mature sperms (SZ) are noticed near the tubular lumen (L).

The histological structure of the control and vitamin C-treated rat testis, observed in toluidine blue-stained semi-thin sections, shows seminiferous tubules with a normal shape and arrangement of spermatogenic cells, resting on an intact basement membrane. Large primary spermatocytes at different stages of meiotic division, round spermatids with acrosomal caps, and numerous mature sperms are present within the tubular lumen (Figures 8A, B, E, F). The CPFX-treated group, examined using toluidine blue-stained semi-thin sections, reveals a reduction in the diameter of seminiferous tubules and the presence of degenerated zones among spermatogenic cells resting on disrupted basement membranes. The tubular lumens are filled with apoptotic spermatozoa. Some early rounded spermatids exhibit disrupted nuclear membranes, and multi-vesicular giant cells with large-sized nuclei are observed near the tubular lumens (Figures 8C, D). The CPFX- and vitamin C-treated rat testis, stained with toluidine blue, shows seminiferous tubules with a normal shape and arrangement of spermatogenic cells resting on an intact basement membrane; large primary spermatocytes at different stages of meiotic division, numerous round spermatids with acrosomal caps, and numerous mature sperms are noticed near the tubular lumen (Figures 8G, H).

### 3.3 Electron microscopic studies

#### 3.3.1 Ultra-thin sections

**FIGURE 9:**
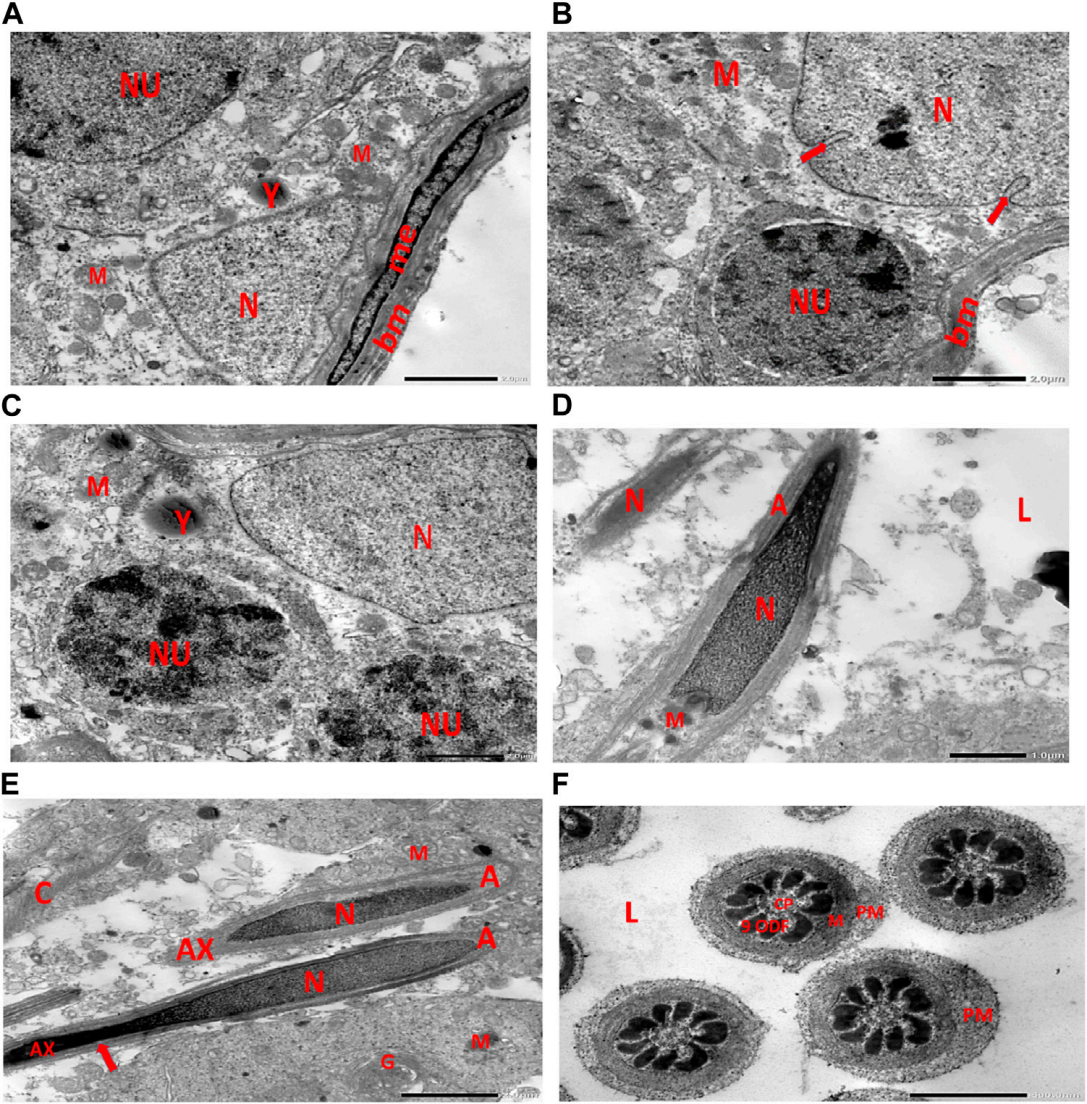
**(A)** Electron micrograph of control rat testis displaying the basement membrane of seminiferous tubules with myoid cell (me) with a flat nucleus. An underlying triangular-shaped SC nucleus (N), rounded mitochondria (M), and lysosomes (Y) are visible. The rounded nucleus (NU) of the primary spermatocyte is also observed. **(B)** Electron micrograph of control rat testis presenting the basement membrane (bm) of seminiferous tubules. Underlying the SC nucleus (N) with indented nuclear membrane (arrows), small rounded mitochondria (M) and spermatogonia with rounded nucleus (NU) are noticed. **(C)** Electron micrograph of the control rat testis displaying SC nucleus (N); and two neighboring primary spermatocytes with rounded mitochondria (M), lysosomes (Y), and rounded nuclei (NU) are noticed. **(D)** Electron micrograph of control rat testis presenting two longitudinally viewed sperms inside the seminiferous tubular lumen (L) with elongated nuclei (N) and overlying acrosomal membrane (A) and middle piece containing mitochondrial helix (M). **(E)** Electron micrograph of control rat testis displaying two longitudinally viewed sperms with acrosomal nuclei (N) and overlying acrosomal membrane (A). The axoneme (AX) is surrounded by a mitochondrial sheath (arrow), numerous mitochondria (M), Golgi (G), and collagen of neighboring spermatids. **(F)** Electron micrograph of the control rat testis showing transverse sections of principal pieces of sperm flagella inside the tubular lumen (L), with a central pair (CP) and plasma membrane (PM) that is closely opposed to the mitochondria (M). The nine outer dense fibers (9 ODFs), regular in shape, are noticed (as shown in Diagram 1A, B).

Electron micrographs of the control rat testis display the basement membrane of the seminiferous tubules, along with a myoid cell characterized by a flat nucleus. Under this layer, a triangular-shaped Sertoli cell nucleus is observed, accompanied by rounded mitochondria and lysosomes. The rounded nucleus of the primary spermatocyte is also visible. Primary spermatocytes display rounded mitochondria, lysosomes, and rounded nuclei. Two longitudinally viewed sperm cells inside the seminiferous tubular lumen exhibit elongated nuclei, an overlying acrosomal membrane, and a middle piece containing the mitochondrial helix. The axoneme is surrounded by a mitochondrial sheath, with numerous mitochondria, Golgi apparatus, and collagen fibers from neighboring spermatids present. Transverse sections of the principal pieces of sperm flagella inside the tubular lumen show a central pair structure, with the plasma membrane closely associated with the mitochondria. The nine outer dense fibers appear regular in shape (Figures 9A–F).

**FIGURE 10:**
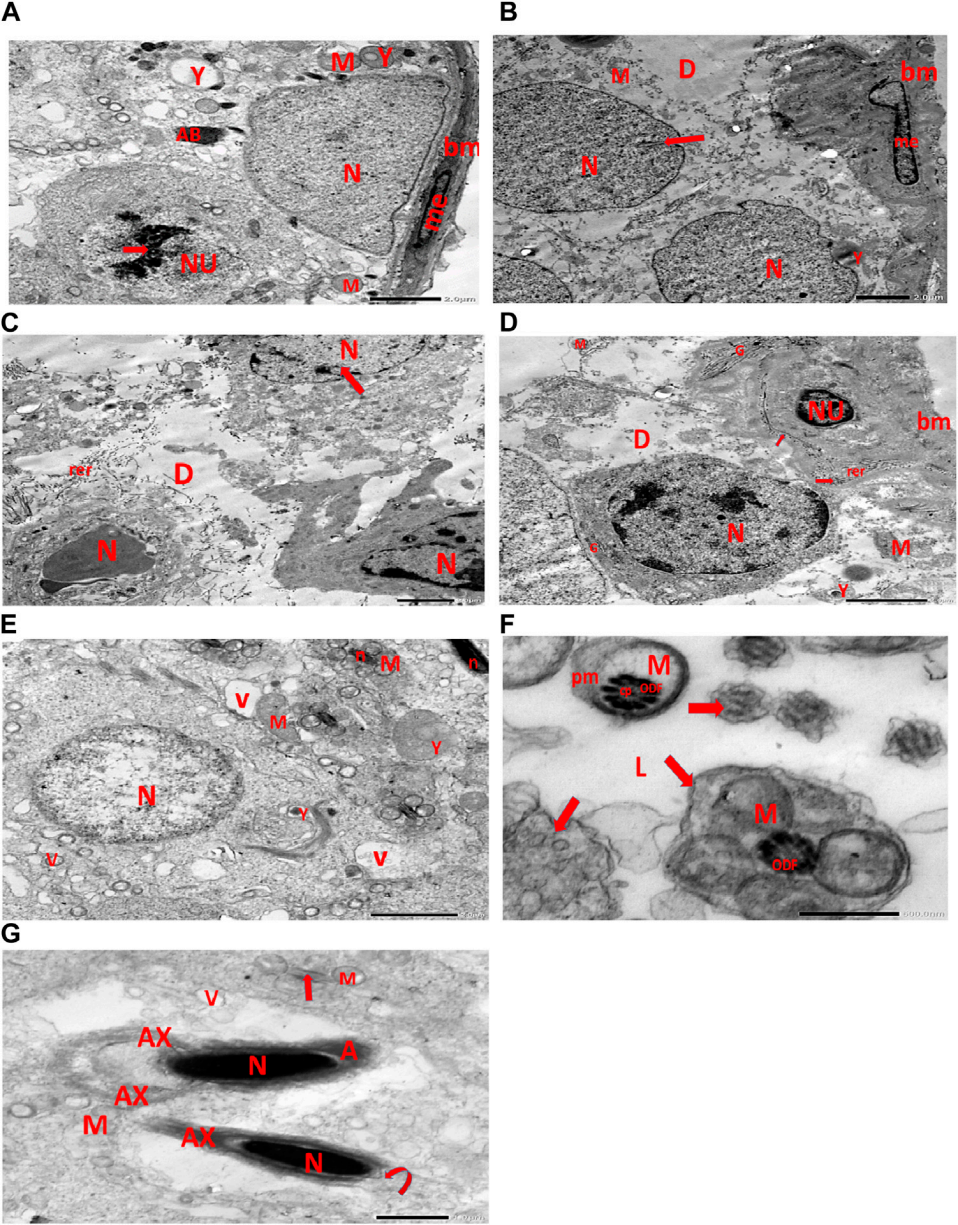
**(A)** Electron micrograph of CPFX-treated rat testis demonstrating the basement membrane of seminiferous tubules with a shrunken myoid cell (me) with the flat elongated nucleus. Underlying the SC, a triangular nucleus (N), rounded mitochondria (M), and lysosomes (Y) are noticed. The rounded nucleus (NU) of the primary spermatocyte with disintegrated crumbled chromatin (pyknosis) (arrow) and an absent nuclear membrane is also observed. **(B)** Electron micrograph of the CPFX-treated rat testis showing the basement membrane of seminiferous tubules with a shrunken myoid cell (me) with a pyknotic hammer-shaped nucleus. Underlying SC nuclei (N), indented nuclear membranes (arrows), small rounded disintegrated mitochondria (M), focal zones of cytoplasmic degeneration (D), where no organelles could be observed, and rounded lysosomes (Y) are present. **(C)** Electron micrograph of CPFX-treated rat testis presenting seminiferous tubules with shrunken spermatogenic cell nuclei (N) with indented nuclear membrane (arrow). Cytoplasmic degeneration (D) and disrupted rough endoplasmic reticulum (rer) are also noticed. **(D)** Electron micrograph of CPFX-treated rat testis displaying the wavy basement membrane (bm) of seminiferous tubules with the underlying spermatogenic cell with a shrunken nucleus (NU), disrupted rough endoplasmic reticulum (rer) with desquamated ribosomes (arrows), and disrupted Golgi shacks (G). Primary spermatocyte with rounded nucleus (N) of primary spermatocyte with a surrounding crumpled Golgi (G), degenerating cytoplasm (D), swollen mitochondria (M), and lysosomes (Y) are observed. **(E)** Electron micrograph of the same group rat testis showing spermatids with nearly rounded nucleus (N), vacuolated cytoplasm (V), and swollen mitochondria (M). Numerous small mitochondria (M) are visible around sperm nuclei (n) and a nearby lysosome (Y). **(F)** Electron micrograph of the same group rat testis showing transverse sections of principal pieces of sperm flagella (arrows) inside the testicular lumen (L), with a central pair (CP) and a loosely packed plasma membrane (PM), which is opposed to the hydropic swollen mitochondria (M). Nine outer dense fibers (9 ODF), atrophic in shape, are noticed. **(G)** Electron micrograph of the same group rat testis presenting two longitudinally viewed short-statured sperms with elongated nuclei (N) and an overlying acrosomal membrane (A). The upper sperm shows bifid axoneme (AX) surrounded with the mitochondrial sheath (M), while the lower sperm exhibits degenerated acrosomal cap (curved arrow). Numerous swollen mitochondria (M) surrounding ill-developed sperm (arrow) are visible.

**FIGURE 11:**
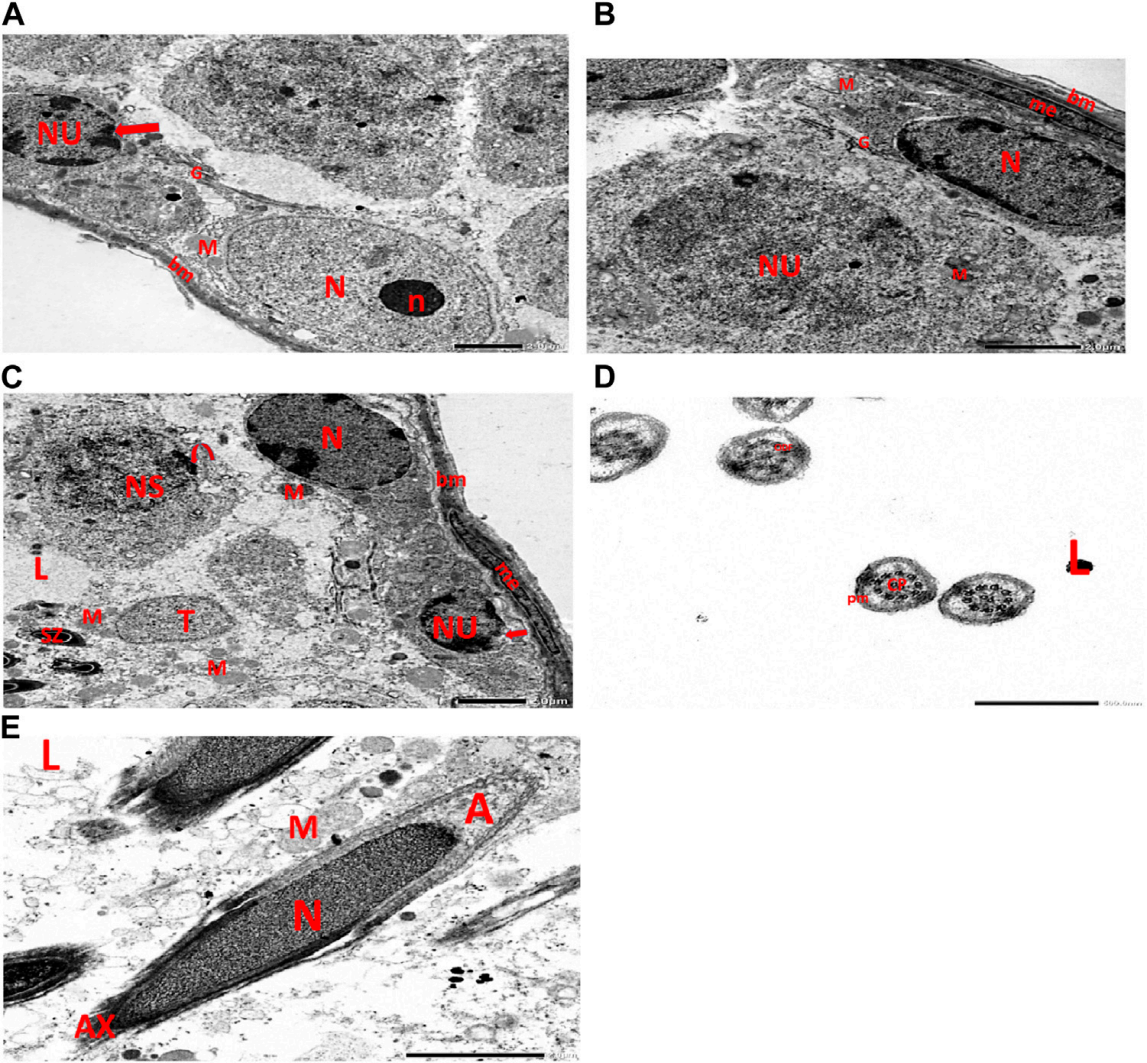
**(A)** Electron micrograph of vitamin C-treated rat testis reveals a well-structured basement membrane surrounding the seminiferous tubules, with underlying spermatogonia exhibiting an oval nucleus (N) and a prominent nucleolus (n), as well as distinct Golgi stacks (G) and rounded mitochondria (M). Sertoli cell is visible with its oval nucleus (NU) and indented nuclear membrane (arrow). **(B)** Another electron micrograph of the same group of rat testis displays a normal basement membrane (bm) surrounding the seminiferous tubules, with myoepithelial cells (me) present along the lumen of the tubules. The basal lamina is populated with Sertoli cells, identified by their elongated nuclei (N) and distinctive cytoplasmic features, including small rounded mitochondria (M) and Golgi stacks (G). Spermatogonia, the immature germ cells, are visible with their round nuclei (NU) and small rounded mitochondria. (M). **(C)** Electron micrograph of the same group rat testis shows a regular basement membrane (bm) of seminiferous tubules with underlying spermatogonia, characterized by oval nuclei (N), Golgi stacks (G), and rounded mitochondria (M). Within this context, primary spermatocytes are identified by their rounded nuclei (NS) and peripheral nucleoli (represented by the curved arrow). These cells also contain oval spermatids (T), which are capped by rounded mitochondria (M). Furthermore, developing sperms (SZ) are observed near the tubular lumen (L). **(D)** Electron micrograph of the same group rat testicular tubular lumen (L) reveals transverse sections of principal pieces of sperm flagella (indicated by arrows). The central pair (CP) is prominently visible, surrounded by the plasma membrane (PM). Additionally, the nine outer dense fibers (9 ODF) are observed, characterized by their regular, distinct shape. **(E)** Electron micrograph of the same group of rat testicular tubular lumens (L) is shown. It displays two longitudinally viewed sperms with elongated nuclei (N) and overlying acrosomal membranes (A). The axoneme (AX) is visible, surrounded by mitochondrial sheaths (M), Golgi (G), and collagen.

**FIGURE 12:**
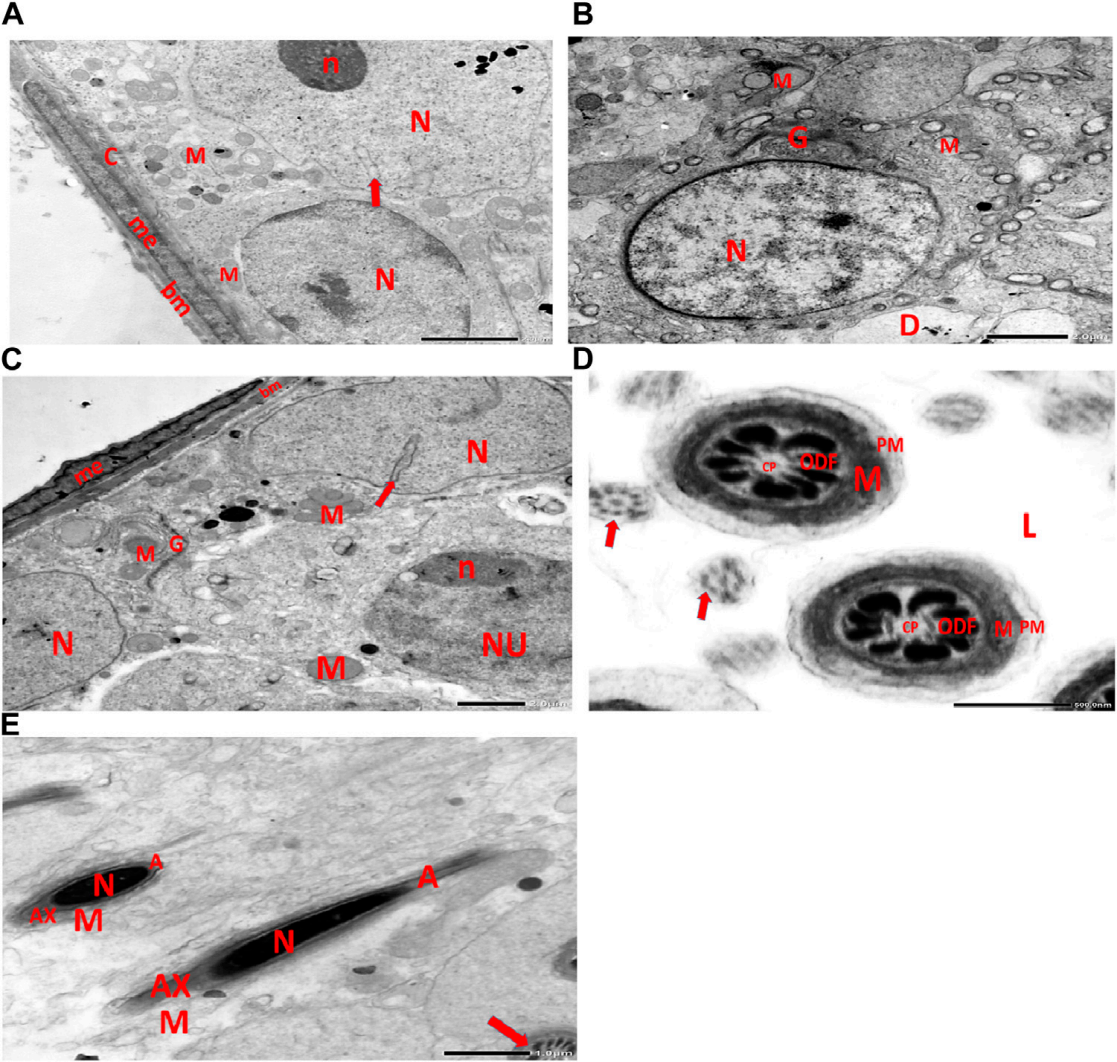
**(A)** This electron micrograph depicts a CPFX- and vitamin C-treated rat testis, displaying a well-structured basement membrane of seminiferous tubules underlain by myoepithelial cells (me) depositing collagen fibers (C). Spermatogonia are visible with oval nuclei (N) and abundant round mitochondria (M). SC is also present, featuring an oval nucleus (N) with an indented nuclear membrane (arrow) and prominent nucleolus (n). **(B)** Another electron micrograph of the same CPFX- and vitamin C-treated rat testis reveals spermatids with round nuclei (N), numerous round mitochondria (M), and well-developed Golgi stacks (G). A degeneration zone (D) is observed neighboring this area. **(C)** This electron micrograph depicts the regular basement membrane (bm) of seminiferous tubules in the testis of a rat, with myoepithelial cells (me) overlying the surface. The underlying structure is a spermatogonia, characterized by an oval nucleus (N) with a prominent nucleolus (n) and rounded mitochondria (M). Additionally, CS is visible, identified by its oval nucleus (N) with an indented nuclear membrane (arrow) and well-developed Golgi apparatus (G) and developing mitochondria (M). **(D)** This electron micrograph presents transverse sections of principal pieces of sperm flagella located within the tubular lumen (L) of the rat testis. The central pair (CP) and the plasma membrane (PM) are clearly visible, and the mitochondria (M) are closely opposed to the plasma membrane. Nine outer dense fibers (9 ODF) are evident, showing regular shapes. Furthermore, small developing sperm flagella (arrows) are discernible. The micrograph illustrates the intricate details of the sperm flagella within the testis of the rodent. **(E)** Electron micrograph of the same group of rat testis presenting two longitudinally viewed sperms with elongated nuclei (N) and an overlying acrosomal membrane (A). The axoneme (AX) is surrounded by a mitochondrial sheath (M), and numerous mitochondria (M) are visible. The Golgi (G) and cross-sectioned sperm flagella (arrow) are also present.

Electron micrographs of the CPFX-treated rat testis reveal a basement membrane of seminiferous tubules with a shrunken myoid cell with a flat, elongated nucleus. Beneath this, an SC with a triangular nucleus is observed, along with rounded mitochondria and lysosomes. The primary spermatocyte exhibits a rounded nucleus with disintegrated, crumbled chromatin (pyknosis) and an absent nuclear membrane. Shrunken spermatogenic cell nuclei with indented nuclear membranes, cytoplasmic degeneration, and disrupted rough endoplasmic reticulum are also noticed. Spermatids with a nearly rounded nucleus, vacuolated cytoplasm, and swollen mitochondria are clearly observed. Transverse sections of principal pieces of sperm flagella inside the testicular lumen show a central pair and a loosely packed plasma membrane, which is opposed to the hydropic, swollen mitochondria. The nine outer dense fibers appear atrophic in shape. Two longitudinally viewed short-statured sperms with elongated nuclei and overlying acrosomal membranes are observed. The upper sperm exhibits a bifid axoneme surrounded by a mitochondrial sheath, while the lower sperm exhibits a degenerated acrosomal cap. Numerous swollen mitochondria surrounding ill-developed sperm are also visible (Figures 10A–G). The electron microscopy of the vitamin C-treated group testis closely resembles that of the control group (Figures 11A–E). In the CPFX- and vitamin C-treated rat testis, a well-structured basement membrane of seminiferous tubules is observed, underlain by myoepithelial cells laying down collagen fibers. Spermatogonia with oval nuclei and abundant round mitochondria are visible. SCs are also present, featuring an oval nucleus with an indented nuclear membrane and a prominent nucleolus. Spermatids exhibit round nuclei, numerous round mitochondria, and well-developed Golgi stacks. Two longitudinally viewed sperms with elongated nuclei and overlying acrosomal membranes are evident. The axoneme is surrounded by a mitochondrial sheath with numerous mitochondria. The Golgi and cross-sectioned sperm flagella are clearly visible (Figures 12A–E).

#### 3.3.2 Control group

Figures 9A–F

#### 3.3.3 CPFX-treated group

Figures 10A–G

#### 3.3.4 Vitamin C-treated group

Figures 11A–E

#### 3.3.5 CPFX- and vitamin C-treated group

Figures 12A–E

### 3.4 Morphometric results

#### 3.4.1 Severity of germ cell degeneration or depletion of seminiferous and epididymal tubules

Table 1, Chart 1

**CHART 1:**
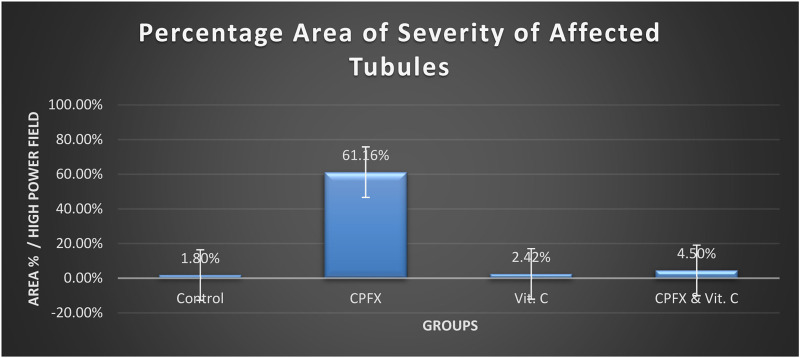
Severity of germ cell degeneration or depletion of seminiferous and epididymal tubules.

#### 3.4.2 Epithelial height of seminiferous tubules

**CHART 2:**
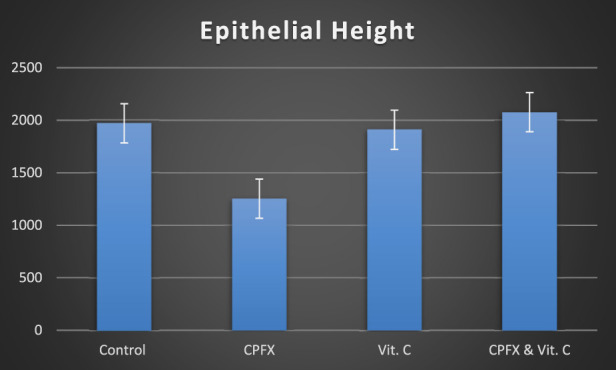
Epithelial height (µm) n = 10.

A significant reduction in the height of seminiferous tubules was observed in the CPFX-treated group (group II) compared to the control group (group I) (p = 0.001), the vitamin C-treated group (group III) (p = 0.001), and the vitamin C- and CPFX-treated group (group IV) (p = 0.001) (Table 2; Chart 2).

### 3.5 Hormonal assay

Chart 3

**CHART 3:**
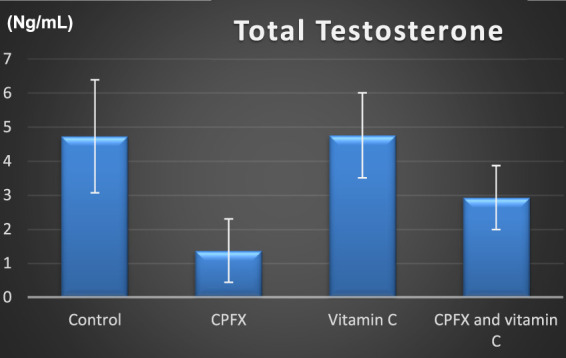
Total testosterone (ng/mL).

**CHART 4:**
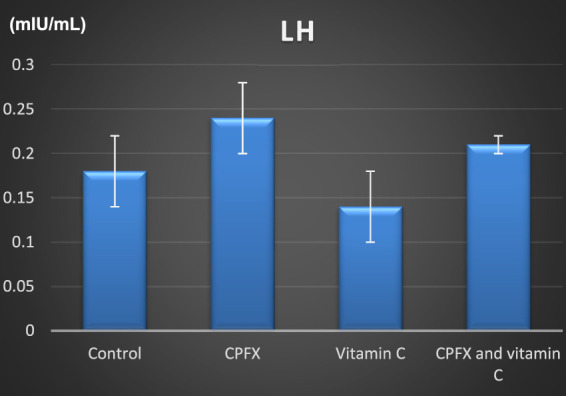
Serum luteinizing hormone (mIU/mL).

#### 3.5.1 Serum luteinizing hormone level

The luteinizing hormone (LH) level was elevated in both the CPFX and CPFX- and vitamin C-treated groups due to the negative feedback mechanism of the hypothalamic–pituitary axis. Decreased testosterone levels stimulate the anterior pituitary to release more LH. Significant results were observed between the CPFX-treated group and the vitamin C-treated group (p = 0.003).

#### 3.5.2 Serum follicle-stimulating hormone (FSH) level

Chart 5

**CHART 5:**
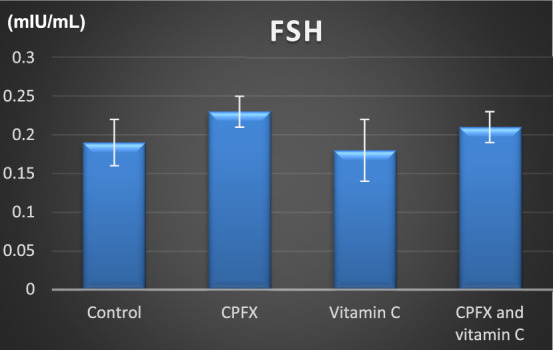
FSH (mIU/mL). * indicates significant *p*-value.

### 3.6 Oxidative stress and antioxidant parameters

#### 3.6.1 Impact of vitamin C treatment on testicular oxidative stress biomarkers nnduced by CPFX

**CHART 6:**
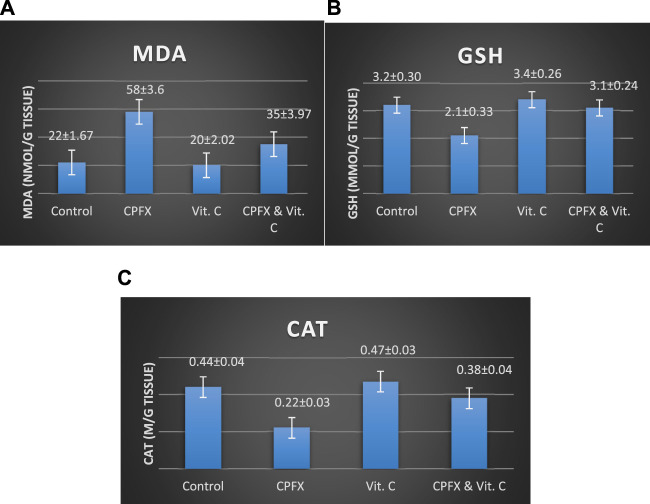
Influence of vitamin C treatment on testicular oxidative stress biomarkers induced by CPFX. **(A)** MDA, **(B)** GSH, and **(C)** CAT. Data are presented as the mean ± SE (n = 10). A *p*-value <0.05 was considered statistically significant.

CPFX administration led to a significant increase in testicular MDA levels (Chart 6A), indicating heightened lipid peroxidation. Concurrently, it substantially reduced antioxidant levels, as evidenced by a marked decrease in the testicular GSH content (Chart 6B) and CAT enzymatic activity (Chart 6C). Conversely, the co-administration of CPFX with vitamin C significantly lowered testicular MDA content and robustly enhanced the antioxidant status, as demonstrated by a notable increase in GSH content and CAT enzymatic activity relative to the CPFX-treated group (Chart 6A–C).

Effects of vitamin C administration on MDA, GSH, and CAT levels in the CPEX-treated rat:

(A) MDA levels (ng/mL): Statistical analysis was performed using a one-way ANOVA, followed by Tukey’s multiple comparison *post hoc* test (F (3,12) = 7.725) . Significant differences were observed between the vitamin C- and CPEX-treated and CPEX groups (p < 0.05) and between the control and CPEX groups (##p < 0.01). (B) GSH levels (μg/mL) in testis (control, n = 10) [vitamin C (n = 10), CPEX (n = 10), or CPEX + vitamin C (n = 10)]: One-way ANOVA was performed, followed by Tukey’s multiple comparison *post hoc* test (F (3,16) = 26.59). Significant differences were observed between the vitamin C- and CPEX- treated and CPEX groups (**p < 0.001) and between the control and CPEX groups (**p < 0.01).(C) CAT levels (pg/mL) in the testis of control rats [(n = 10), vitamin C (n = 10), or CPEX (n = 10), or CPEX + vitamin C]: One-way ANOVA was performed, followed by Tukey’s multiple comparison *post hoc* test (F (3,15) = 7.300). Significant differences were observed between the control and CPEX groups (*p < 0.05) and between the vitamin C- and CPEX-treated group and the CPEX group (##p < 0.01).

## 4 Discussion

Medical, psychological, and financial implications of infertility can exacerbate stress and distress in individuals. Research into the importance of nutrients and trace elements in male reproductive health, particularly sperm function, fertility, and associated mechanisms, is believed to provide a solid theoretical foundation for developing targeted clinical interventions in infertility (Chao et al., 2022). As oxidative stress impairs sperm function and general reproductive health, it is a major contributing factor to male infertility. This study investigated the potential therapeutic role of vitamin C in treating male infertility. Evidence suggests that each antioxidant alone can improve sperm health and reproductive outcomes (Kaltsas, 2023).

As CPFX, a broad-spectrum antibiotic frequently prescribed for genitourinary tract infections, can have adverse effects on male reproductive organs by traversing the seminal fluid, its mechanism of action involves inhibiting DNA gyrase and topoisomerase, impeding bacterial DNA replication (Sharma et al., 2010; Shrinivas and Revanasiddappa, 2015).

In the current rat model study, conducted over a 60-day period post-CPFX administration, reversible testicular damage induced by oral CPFX treatment was observed. Examples of this damage include blood vessel congestion and bleeding near seminiferous tubules, accompanied by peritubular edema. Significant necrosis of spermatogonia cells lining seminiferous tubules initiated apoptotic pathways and resulted in a decrease in sperm count, disrupting cellular organization (Elbetieha and Da’as, 2003; Celik-Ozenci et al., 2011; Moghimi et al., 2021).

In the same CPFX-treated rats, the ultrastructure revealed a shrunken basement membrane of seminiferous tubules with myoid cells exhibiting flat, elongated nuclei. The nuclei of primary spermatocytes were rounded, displaying disintegrated chromatin, cytoplasmic degeneration, and characteristic apoptotic figures indicative of apoptotic cell death. Disrupted rough endoplasmic reticulum was also present (Zhang et al., 2020). Apoptosis, a highly regulated process of cell death essential for maintaining the balance between cell proliferation and differentiation, was observed in the form of increased electron density in gonocytes, referred to as dark cells. These cells exhibited blebbing of the plasma membrane, mitochondrial swelling, and distention of endoplasmic reticula, suggesting an apoptosis-like and hypoxia-related type of cell degeneration. Normal cell turnover, healthy immune system formation and operation, hormone-dependent atrophy, embryonic development, and chemically induced cell death are all thought to depend on apoptosis (Leist and Jaattela, 2001; Elmore, 2007; Schenk et al., 2017). This was measured by severity grade of percentage of tubules affected as described by Lanning et al. (2002) as follows: grade 1 (minimal) <5% of tubules affected, grade 2 (slight) 5–25% tubules affected, grade 3 (moderate) 25–50% tubules affected and grade 4 (marked) 50–75% tubules affected 5 (severe) >75% tubules affected as in Table 1. The current ultrastructural findings of disrupted rough endoplasmic reticulum and mitochondrial demises in the CPFX-treated group are also in accordance with the findings of Jomova et al. (2024), who emphasized that ROS produced in mitochondria (mt-ROS) were formerly regarded to be unwanted byproducts of oxidative metabolism. Several studies have indicated that mitochondrial ROS, especially those essential for autophagy and the immunological response, contribute to cellular signaling. Another key source of ROS is the endoplasmic reticulum, which primarily produces hydrogen peroxide as a byproduct of protein folding. The mitochondria–endoplasmic reticulum interaction areas are referred to as mitochondria-associated membranes (MaMs). MaMs play critical roles in calcium signaling, lipid transport, and cell death; thus, endoplasmic reticulum disruptions influence mitochondria. Disruptions in the endoplasmic reticulum can be caused by increased oxidative stress, saturated fatty acid deposition, hypoxia, or other stimuli, resulting in the accumulation of unfolded proteins in the endoplasmic reticulum lumen, which is filled with proteins, antioxidants, and essential minerals. In the testes of rats treated with CPFX and vitamin C, observations revealed a regular basement membrane of seminiferous tubules, with elongated myoepithelial cells depositing collagen fibers. As described by Shokri et al. (2012), the BM contacts the seminiferous epithelium and contains laminin and collagen types I and IV, synthesized by Sertoli cells. The middle zone, known as the myoid layer, comprises the first three to five incomplete layers of modified smooth muscle cells called myoid cells. According to the ultrastructural findings of the current study, these cells are located within the extracellular matrix among fibrillary lamellar structures composed of collagen and laminin in the normal testis. Myoid cells contain numerous actin, myosin, and desmin filaments. Spermatogonia with oval nuclei and numerous rounded mitochondria were also observed, along with SCs with oval nuclei and indented nuclear membranes. Transverse sections of principal pieces of sperm flagella within the tubular lumen showed a central pair and closely opposed plasma membrane to the mitochondria. Regularly shaped nine outer dense fibers were also noted. All these alterations culminated in a decrease in male fertility, as evidenced by significant reductions in seminiferous tubular epithelial heights and diameters observed in CPFX-treated rat testes, as depicted in Tables 1, 2. Due to the presence of germinal lineage cells at various phases of the seminiferous cycle within different tubular segments, the diameters of seminiferous tubules varied in cross sections. In the testes of treated rats, a reduction in tubular size was associated with the separation and loss of germ cells due to cell death. Caneguim et al. (2009), Mathur and D' Cruz (2011), and Adamczewska et al. (2022) reported that degeneration in spermatogenic cells was correlated with reductions in sperm count and motility, ultimately leading to spermatogenic failure. In summary, the results of this study suggested that CPFX has toxic effects on testicular physiological function, which correspond well with histopathological changes. However, the antioxidant vitamin C can improve testicular function by alleviating oxidative stress induced by CPFX. As a potent water-soluble antioxidant, vitamin C likely mitigates drug-induced oxidative stress by scavenging generated free radicals (Ziamajidi et al., 2023). This is consistent with our findings; oxidative stress is a critical factor in CPFX-induced testicular damage. Upon exposure to CPFX, ROS production is stimulated, leading to membrane damage, increased lipid peroxidation, and, ultimately, atrophy in seminiferous tubules and apoptosis of spermatocytes (Osawe and Farombi, 2013). Although follicle-stimulating hormone (FSH) and testosterone exhibit overlapping and synergistic effects on spermatogenesis, the role of testosterone is more prominent in supporting spermatid differentiation, whereas FSH plays a crucial role in supporting spermatogonia numbers and differentiation (Shetty et al., 2006). Researchers observed a decrease in serum LH, which they attributed to reduced gonadotropin-releasing hormone (GnRH) stimulation, resulting in decreased testosterone levels in infected animals (Tables 3, 4) (Charts 4, 5). Additionally, elevated estradiol levels and decreased testosterone levels were observed, along with reduced sperm count and increased abnormal sperm index (Faccio et al., 2014; Lin et al., 2020). A significant decrease in testosterone levels (*p* ≤ 0.05) (Table 5, Chart 3) aligns with the findings of Abubakar et al. (2016), supporting the concept that pollution exerts toxic effects on the hypothalamic or supra-hypothalamic sites, while leaving the pituitary–testicular axis intact. Although their study showed no significant effect on LH levels, it demonstrated a remarkable decrease in testosterone levels. Daku et al. (2016) contended that heavy metals target spermatogenesis and sperm within the epididymis, leading to reproductive toxicity, rather than affecting the hypothalamic–pituitary–testicular (H-P-T) axis. They stated that the gonadotoxic effects are localized within the testes, having minimal influence on hormonal status and remaining inactive at extratesticular sites. Xie et al. (2019) showed that the testis debris-eliminating and regenerative action in the animals with CPFX-induced testicular damage was primarily driven by the testis-protective, anti-inflammatory, and antioxidant effects of the treatment. These effects were leveraged to the ability of the compound to decrease oxidative stress, enhance antioxidant status, maintain the stability of antioxidant protection systems, and minimize the formation of oxidative and inflammatory products. The findings of Moradi et al. (2023) coincide with our findings, showing that vitamin C restored spermatogenesis by improving sperm viability, morphology, and chromatin integrity. Testosterone levels and testes histopathology were significantly improved in the vitamin C-administrated groups. Additionally, the treatment of vitamin C significantly reduced MDA levels, increased total antioxidant capacity (TAC) levels, and mitigated oxidative damage. Vitamin C administration was found to be a helpful therapeutic against drug-associated reproductive toxicity and male sub/infertility since it dramatically decreased oxidative stress and apoptosis. In the present study, CPFX exposure significantly increased the content of MDA, a product of lipid peroxidation, while markedly depleting the antioxidant defense system, as evidenced by reduced GSH levels and CAT activity. Conversely, CAT and GSH levels significantly increased in vitamin C- and CPFX-treated rats compared to CPFX-treated rats, while MDA levels decreased in a statistically significant manner. The findings of this research highlight the role of oxidative stress in CPFX-induced testicular damage and suggest that antioxidant therapy may be a potential strategy for mitigating the toxic effects of CPFX on male fertility. This is consistent with findings of Gehin et al. (2006), who confirmed that antioxidant enzymes like catalase, superoxide dismutase (SOD), glutathione-peroxidase (GSH-Px), and glutathione-reductase (GSSG-Red) play a critical role in enzymatic defense against ROS; they also emphasized that intracellular reduced GSH is also crucial. They also determined that vitamin C or vitamin E supplements may help make up for oxidative damage in defending cells against this attack. Moreover, Yadegari et al. (2024) reported that adding vitamin C to acrylamide (ACR)-treated rats exhibited mild degeneration and rupture in testicular and epididymal epithelium integrity.

**TABLE 1:** A semiquantitative grading system to record severity of germ cell degeneration or depletion in seminiferous and epididymal tubules.

Standard severity (score)	1 minimal	2 slight	3 moderate	4 marked	5 severe
Proportion of affected tubules	<5%	5%–25%	25%–50%	50%–75%	>75%
Control group	0.5%–3%	–	–	–	–
CPFX group	–	–	–	50%–70%	–
Vitamin C group	1%–4%	–	–	–	–
CPFX and vitamin C group	–	3%–8%	–	–	–

Severity grade: approximate proportion of tubules affected 1 (minimal) < 5% of tubules affected; 2 (slight) 5%–25% tubules affected; 3 (moderate) 25%–50% tubules affected; 4 (marked) 50%–75% tubules affected; 5 (severe) > 75% tubules affected.

**TABLE 2:** Epithelial height (semi-thin sections): n = 10.

Epithelial height of seminiferous tubule (µm)		Group I	Group II	Group III	Group IV
Range		1,318.07–1804.07	967.24–1,223.22	1,418.07–1,799.03	1,304.07–1,722.03
Mean ± SD		1,588.52 ± 154.06	1,066.23 ± 73.33	1,608.52 ± 129.53	1,565.53 ± 121.38
F test		45.030			
*p*-value		0.001*			

Group I vs. Group II	Group I vs. Group III	Group I vs. Group IV	Group II vs. Group III	Group II vs. Group IV	Group III vs. Group IV
0.001*	0.719	0.679	0.001*	0.001*	0.440

*p*-value is significant (*). vs.: versus.

**TABLE 3:** Alterations in the levels of serum follicle-stimulating hormone (FSH; mIU/mL) in rats in various groups under study. Data are presented as the mean ± SE (n = 10). A *p*-value <0.05 was considered statistically significant.

FSH	Control	CPFX		Vitamin C	CPFX and vitamin C
Range	0.15–0.22	0.2–0.25		0.13–0.23	0.19–0.23
Mean ± SD	0.19 ± 0.03	0.23 ± 0.02		0.18 ± 0.04	0.21 ± 0.02
F test	2.579				
*p*-value	0.057				

Control vs. CPFX	Control vs. vitamin C	Control vs. CPFX and vitamin C	CPFX vs. vitamin C	CPFX vs. CPFX and vitamin C	Vitamin C vs. CPFX and vitamin C
0.092	0.966	0.664	0.058	0.527	0.399

Insignificant value is observed.

**TABLE 4:** Alterations in the levels of serum luteinizing hormone (LH; mIU/mL) in rats of various groups under study**.** Data are presented as the mean ± SE (n = 10). A *p*-value <0.05 was considered statistically significant.

LH	Control	CPFX		Vitamin C	CPFX and vitamin C
Range	0.13–0.24	0.2–0.29		0.11–0.20	0.19–0.22
Mean ± SD	0.18 ± 0.04	0.24 ± 0.04		0.14 ± 0.04	0.21 ± 0.01
F test	6.587				
*p*-value	0.004*				

Control vs. CPFX	Control vs. vitamin C	Control vs. CPFX and vitamin C	CPFX vs. vitamin C	CPFX vs. CPFX and vitamin C	Vitamin C vs. CPFX and vitamin C
0.090	0.342	0.646	0.003*	0.538	0.045*

**TABLE 5:** Alterations in the levels of total serum testosterone (ng/mL) in rats in various groups under study. Data are presented as the mean ± SE (n = 10). A *p*-value <0.05 was considered statistically significant.

Total Testosterone	Control	CPFX		Vitamin C	CPFX and vitamin C
Range	3.2–7.5	0.35–2.5		3.20–6.5	2.11–4.5
Mean ± SD	4.73 ± 1.66	1.37 ± 0.93		4.76 ± 1.25	2.96 ± 0.94
F test	8.711				
*p*-value	0.003*				

Control vs. CPFX	Control vs. Vitamin C	Control vs. CPFX and vitamin C	CPFX vs. Vitamin C	CPFX vs. CPFX and vitamin C	Vitamin C vs. CPFX and vitamin C
0.003*	0.998	0.147	0.002*	0.213	0.137

*p-*value is significant (*).

Serum testosterone was significantly reduced in the CPFX-treated versus (vs.) the control group (p = 0.003). In addition, significant results were shown between the CPFX vs. vitamin C-treated group (*p* = 0.002).

## 5 Conclusion

The study found that vitamin C has a protective effect against testicular and epididymal damage caused by CPFX in adult male albino rats. This suggests that vitamin C could potentially be used as a therapeutic intervention for various cognitive deficits. The research further indicates that CPFX-induced testicular toxicity in rat models may be reversible, particularly when combined with vitamin C, which promotes the regeneration of the spermatogenic epithelium.

## Data Availability

The raw data supporting the conclusions of this article will be made available by the authors, without undue reservation.
